# The Potential Protective Effects of EGCG Against Epilepsy‐Induced Damage in Rats by Mitigating Oxidative Stress, Inflammation, and Apoptosis

**DOI:** 10.1155/sci5/8209714

**Published:** 2025-12-22

**Authors:** Sarah Alatawi, Manal S. Albalawi, Ruba M. Alfaifi, Rand Al-Twalhy, Manal D. Al-Johani, Danah Alanazi, Rinad M. Al-Otaibi, Hanan M. Hassan, Mohammed M. H. Al-Gayyar

**Affiliations:** ^1^ Faculty of Pharmacy, University of Tabuk, Tabuk, Saudi Arabia, ut.edu.sa; ^2^ Department of Pharmacology and Biochemistry, Faculty of Pharmacy, Delta University for Science and Technology, Gamasa City, Egypt, deltauniv.edu.eg; ^3^ Department of Biochemistry, Faculty of Pharmacy, Mansoura University, Mansoura, Egypt, mans.edu.eg; ^4^ Department of Pharmaceutical Chemistry, Faculty of Pharmacy, University of Tabuk, Tabuk, Saudi Arabia, ut.edu.sa

**Keywords:** acetylcholinesterase (AChE), B-cell lymphoma 2 (BCL2), Bcl-2-associated X protein (BAX), dopamine (DA), heme oxygenase-1 (HO-1), interleukin-1 beta (IL-1β), nuclear factor 2 (Nrf2), nuclear factor kappa B (NF-κB), tumor necrosis factor alpha (TNF-α)

## Abstract

We conducted this study to evaluate the protective effects of Epigallocatechin‐3‐gallate (EGCG) against epilepsy in rats, with a specific focus on its potential to mitigate oxidative stress, inflammation, and apoptosis. Epilepsy was induced in rats using pentylenetetrazol (PTZ), followed by treatment with 20 mg/kg of EGCG. The effects of EGCG were assessed on seizure severity and frequency, as well as acetylcholinesterase (AChE) activity. Brain sections were stained with cresyl violet and immune‐stained with anti‐Nrf2 antibody. Furthermore, expressions and concentrations of B‐Cell Lymphoma 2 (BCL2), Nuclear Factor Erythroid 2–Related Factor‐2 (Nrf2), nuclear factor κB (NFκB), BCL2‐associated X (BAX), tumor necrosis factor‐α (TNF‐α), and Interleukin‐1 β (IL‐1β) in brain tissues were analyzed. Rats showed significant behavioral improvement following EGCG treatment. Analysis of the dentate gyrus sections demonstrated a modest increase in the staining intensity of Nissl granules after EGCG. Additionally, EGCG was observed to increase the expression levels of BCL2, Nrf2, and Heme Oxygenase‐1 (HO‐1), while concurrently reducing the expression of BAX, NF‐κB, TNF‐α, and IL‐1β. In conclusion, EGCG demonstrates protective effects against epilepsy. The underlying mechanisms may be attributed to its capacity to increase antioxidant activity by the upregulation of Nrf2 and HO‐1. EGCG appears to mitigate inflammation by downregulating NF‐κB, TNF‐α, and IL‐1β, thereby decreasing cellular apoptosis through the downregulation of BAX and upregulation of BCL‐2.

## 1. Introduction

Epilepsy, a neurological disorder, is characterized by recurrent seizures, which arise from sudden, abnormal electrical activity in the brain, leading to transient disruptions in normal brain function. These seizures can manifest in diverse forms, such as convulsions, loss of awareness, unusual sensations, and uncontrollable movements [1]. The mechanisms underlying epilepsy involve the complex interplay of several key neurotransmitters, including serotonin, dopamine, γ‐aminobutyric acid (GABA), glutamate, and noradrenaline. These neurotransmitters modulate the balance of excitation and inhibition in the brain, influencing the onset and spread of seizures. Symptoms of epilepsy exhibit considerable variability and may encompass transient confusion, loss of consciousness, unusual olfactory or gustatory perceptions, and sudden unexplained emotional states [2]. Epilepsy impacts around 50 million individuals worldwide. The prevalence of active epilepsy at any given time was 6.38 per 1000 people, with a lifetime prevalence rate of 7.60 per 1000 people. The annual cumulative incidence of epilepsy was reported at 67.77 per 100,000 individuals, and the incidence rate stood at 61.44 per 100,000 person‐years. The prevalence of epilepsy remained consistent across different age groups, genders, and levels of study quality [3].

An array of treatment modalities for the management of epilepsy encompass the application of medications that target specific cerebral structures, particularly those associated with the neurotransmitter GABA. Valproic acid, pregabalin, and other drugs that modulate glutamate release, such as lamotrigine, are among these medications. Furthermore, medications acting on neuronal voltage‐dependent sodium channels, such as phenytoin, topiramate, and carbamazepine, are frequently utilized in epilepsy management [4]. It is imperative to recognize that while these drugs effectively control seizures, they are also associated with considerable side effects. These adverse effects may encompass the depression of the central nervous system and respiratory function, cardiovascular complications, gastrointestinal issues, hematological complications, increased susceptibility to specific malignancies, hepatic impairments, hypersensitivity reactions, heightened vulnerability to infections, and potential for localized hemorrhaging [5]. Hence, it is crucial to continue pursuing novel therapeutic agents that can mitigate or minimize these adverse effects.

During epileptic seizures, there is a pathological surge in neuronal excitability that results in excessive neuronal firing. This hyperactivity leads to a significant increase in brain oxygen consumption, which, in turn, disrupts mitochondrial respiratory chain function. Consequently, there is a massive overproduction of reactive oxygen species (ROS), such as superoxide and hydrogen peroxide [6]. Simultaneously, the body’s antioxidant defense mechanisms become overwhelmed and are often suppressed. Key antioxidant enzymes, including superoxide dismutase (SOD), catalase, and glutathione peroxidase, become less effective in neutralizing the excess ROS. This suppression creates a critical imbalance between oxidative stress and the body’s antioxidant capacity, tipping the scales toward oxidative damage [7]. Excessive ROS can directly harm neuronal cellular structures, leading to lipid peroxidation, protein denaturation, and DNA damage. Moreover, ROS are known to compromise the integrity of the blood–brain barrier (BBB), which serves as a protective filter for the central nervous system. This disruption can facilitate the entry of neurotoxic substances and inflammatory mediators into the brain’s microenvironment [8]. In addition, ROS promotes the release of pro‐inflammatory cytokines, such as Interleukin‐1β (IL‐1β) and tumor necrosis factor‐α (TNF‐α), which further intensify neuroinflammation and exacerbate mitochondrial dysfunction. This vicious cycle establishes a self‐perpetuating loop characterized by oxidative stress, leading to neuronal damage, followed by epileptic seizures [6].

Green tea extract displays promising potential as a source for novel molecular treatments. Derived from the dried leaves of the *Camellia sinensis* plant, green tea has been a popular beverage for millennia. Esteemed in Traditional Chinese Medicine for its purported capacity to aid in disease prevention and treatment, its historical use underscores its significance [9]. Epigallocatechin‐3‐gallate (EGCG) is a highly potent and biologically active polyphenol compound categorized as a catechin, a type of flavonoid. This compound is abundant in green tea and its extracts and is well known for its significant bioactivity. Its extensive research and widespread recognition position it as one of the most vital and beneficial compounds found in green tea, with effects including antioxidant, anti‐inflammatory, and anti‐apoptotic properties [10]. The observed therapeutic benefits in treating lithium–pilocarpine‐induced epilepsy were attributed to the suppression of the TLR4/NF‐κB signaling pathway. This suppression yielded favorable results in the management of the condition [11]. Therefore, we conducted this study to evaluate the protective effects of EGCG against epilepsy‐induced seizures in rats by inhibiting oxidative stress, inflammation, and apoptosis.

## 2. Materials and Methods

### 2.1. Animals and Treatment Outlines

The research study involved 40 Sprague Dawley rats weighing 180–200 g. The number of rats was calculated using the resource equation method. These rats were housed under controlled laboratory conditions, with a standard temperature maintained to ensure an optimal environment. The lighting schedule adhered to a strict 12‐h light and 12‐h dark cycle. The Research Ethics Committee of the Faculty of Pharmacy at Delta University of Science and Technology approved the animal research protocol, which received the identifier FPDU16/2023. Throughout the study, the rats had unrestricted access to food and water. The rats were divided into four distinct experimental groups, each comprising 10 rats. The rats and cages are assigned to a coding system. All treatments administered to the rats were conducted by a member of the research team who was unaware of which treatment each animal received. These treatments were applied in the same order and during the same time period each morning.

Control group. The rats received no treatment throughout the experiments.

Control group was treated with EGCG. Rats received 20 mg/kg of EGCG (Sigma‐Aldrich Chemicals Co., St. Louis, MO, USA) daily by oral gavage for 3 weeks.

Epilepsy group. Rats were administered 35 mg/kg of pentylenetetrazol (PTZ) intraperitoneally three times weekly for 3 weeks.

EGCG‐treated epilepsy rat group. After the rats were induced with epilepsy, they were given 20 mg/kg of EGCG daily via oral gavage for 3 weeks.

### 2.2. Evaluation of Seizures

The responses triggered by PTZ were meticulously documented by two impartial observers who were kept unaware of the experimental conditions. Seizures were purposefully initiated between 8:00 and 10:00 a.m. to minimize potential disruption from the animals′ circadian rhythms. The severity of the seizures was evaluated using the following scale: (0) Absence of Further Seizures; (1) a Generalized Seizure; (2) a Generalized Seizure Accompanied by the Loss of Righting Reflex; (3) a Generalized Seizure with Loss of Righting Reflex and Simultaneous Running and Bouncing; and (4) All Behaviors Associated with a Score of 3 Plus Forelimb Tonus.

### 2.3. Sample Collection

The brain was dissected entirely, measured, and weighed. A section of the excised brain was preserved in 10% buffered formalin and utilized for morphological and immunohistochemical analysis. The remaining portion was homogenized in a tenfold volume of ice‐cold sodium–potassium phosphate buffer (0.01 M, pH 7.4) containing 1.15% KCl. The resulting supernatant was then frozen at −80°C.

### 2.4. Morphologic Analysis and Immunohistochemistry

The brain tissue, initially preserved in 10% formalin, underwent meticulous paraffin encasement to ensure long‐term stability and integrity. Once embedded, the tissue was sectioned at 5 μm to facilitate a detailed examination of its structural composition. These sections were subsequently stained with cresyl violet, a dye that highlights the neuronal structures. To ensure the validity and reliability of observations on stained sections, rigorous, anonymized assessments were conducted to minimize potential bias during evaluation. Following this initial assessment, finer 5‐μm‐thick sections were crafted from the paraffin blocks containing the brain tissues. Immunostaining was then performed on these thinner sections using monoclonal anti‐Nrf2 antibodies from MyBioSource, Inc., in San Diego, CA, USA, at 4°C. Following the primary antibody incubation, the sections received treatment with a horseradish peroxidase–conjugated secondary antibody. To further enhance contrast and delineate cellular structures, the sections were counterstained with hematoxylin [12–14].

### 2.5. Enzyme‐Linked Immunosorbent (ELISA) Assay

ELISA kits obtained from MyBioSource, Inc. (San Diego, CA, USA), were utilized to evaluate B‐Cell Lymphoma 2 (BCL2), BCL2‐associated X (BAX), Heme Oxygenase‐1 (HO‐1), IL‐1β, Nuclear Factor Erythroid 2–Related Factor‐2 (Nrf2), and TNF‐α following the manufacturer’s guidelines.

### 2.6. Quantitative Real‐Time Polymerase Chain Reaction (qRT‐PCR)

The mRNA expression levels of key regulatory factors, including nuclear factor κB (NFκB), BCL2, BAX, HO‐1, IL‐1β, Nrf2, and TNF‐α, in rat brain lysates were quantified using established methodologies developed by our research team. Validated gene‐specific PCR primers, listed in Table 1, were used for this analysis. β‐actin was employed as the housekeeping gene and internal reference control to ensure accuracy [15–17].

**Table 1:** The primers set used for the detection of gene expression in rats.

Name	Sequence		Reference sequence
β‐actin	Forward	5′‐CCC​GCG​AGT​ACA​ACC​TTC​TT‐3′	NM_031144.3
	Reverse	5′‐CTA​GTC​TCC​CTC​CCT​CAG​G‐3′	

NFκB	Forward	5′‐TCT​GTT​TCC​CCT​CAT​CTT​TCC‐3′	NM_199267.2
	Reverse	5′‐GGG​TGC​GTC​TTA​GTG​GTA​TCT​G‐3′	

TNF‐α	Forward	5′‐AAA​TGG​GCT​CCC​TCT​CAT​CAG​TTC‐3′	NM_012675.3
	Reverse	5′‐TCT​GCT​TGG​TGG​TTT​GCT​ACG​AC‐3′	

IL‐1β	Forward	5′‐CAC​CTC​TCA​AGC​AGA​GCA​CAG‐3′	NM_031512.2
	Reverse	5′‐GGG​TTC​CAT​GGT​GAA​GTC​AAC‐3′	

Nrf2	Forward	5′‐CTC​TCT​GGA​GAC​GGC​CAT​GAC​T‐3′	NM_001399173.1
	Reverse	5′‐CTG​GGC​TGG​GGA​CAG​TGG​TAG​T‐3′	

HO‐1	Forward	5′‐CAC​CAG​CCA​CAC​AGC​ACT​AC‐3′	NM_012580.2
	Reverse	5′‐CAC​CCA​CCC​CTC​AAA​AGA​CA‐3′	

BCL2	Forward	5′‐ACT​TCT​CTC​GTC​GCT​ACC​GTC​G‐3′	NM_016993.2
	Reverse	5′‐CCC​TGA​AGA​GTT​CCT​CCA​CCA​CC‐3′	

BAX	Forward	5′‐TGG​GCT​GGA​CAC​TGG​ACT​TC‐3′	NM_017059.2
	Reverse	5′‐CTT​CCA​GAT​GGT​GAG​TGA​GGC‐3′	

### 2.7. Statistical Analysis

The results are presented as the mean ± standard error. To assess the normality of the sample distribution, we utilized the Kolmogorov–Smirnov (K–S) test. To compare differences between groups, we employed a one‐way analysis of variance (ANOVA) followed by a post hoc Bonferroni correction test, which helps to control for Type I errors when multiple comparisons are made. All statistical analyses were conducted using SPSS Version 20, a robust statistical software developed by IBM, based in Armonk, NY, USA. Throughout the analysis, we defined statistical significance as a *p* value of less than 0.05.

## 3. Results

### 3.1. Antiepileptic Activities of EGCG

In the study, epileptic rats exhibited a significant increase in the seizure stage and frequency of spontaneous recurrent seizures compared to the control group. Furthermore, the epileptic rats showed a 56.77% reduction in hippocampal dopamine concentrations and a 1.97‐fold increase in hippocampal acetylcholinesterase (AChE) activity compared to the control rats. However, treatment of the epileptic rats with EGCG resulted in a 60.61% reduction in the seizure stage and a 58.09% decrease in the frequency of spontaneous recurrent seizures compared to the untreated epileptic rats. Additionally, EGCG treatment led to a 1.77‐fold increase in hippocampal dopamine and a 36.99% reduction in hippocampal AChE activity compared to the untreated epileptic rats (Figure 1).

Figure 1Effect of epilepsy and 20 mg/kg EGCG on the seizure stage (a), frequency of spontaneous recurrent seizures (b), hippocampus dopamine concentration (c), and hippocampus AChE activity (d). AChE, acetylcholine esterase; EGCG, Epigallocatechin‐3‐gallate.

**Figure:**
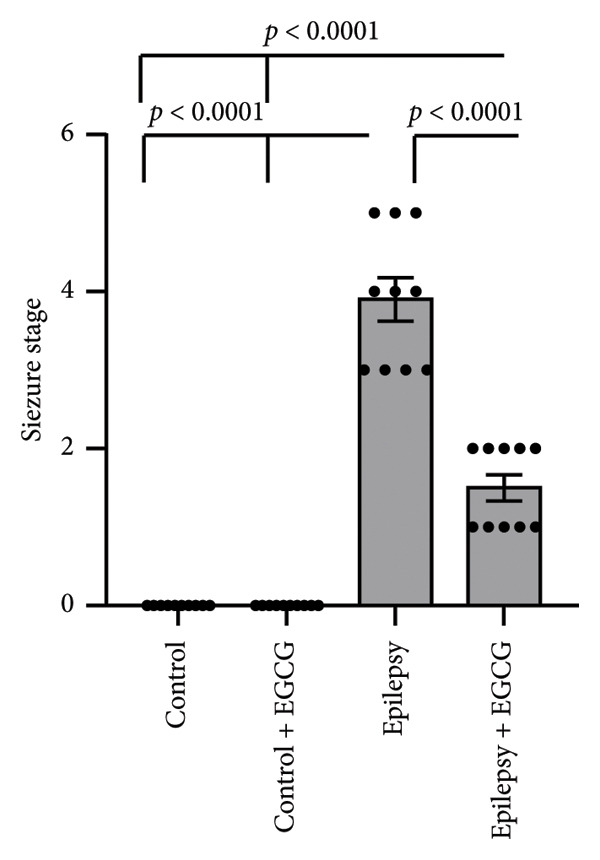
(a)

**Figure:**
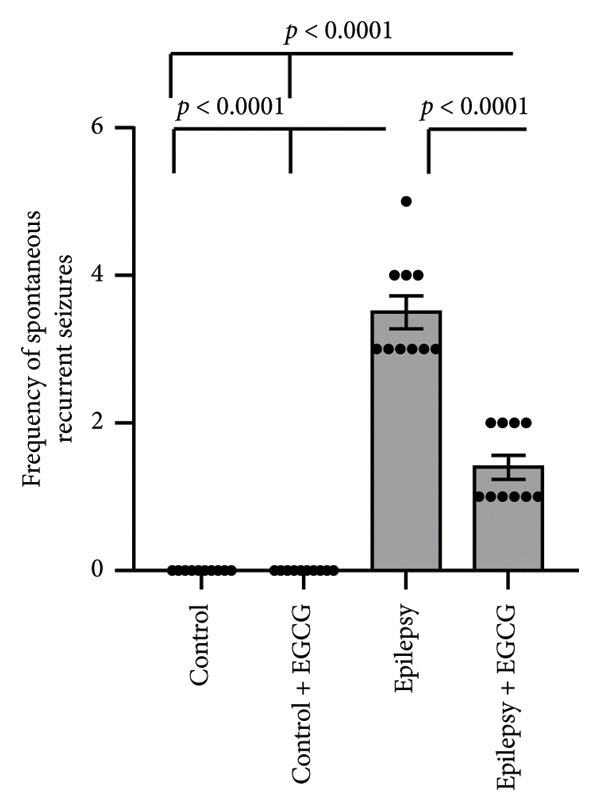
(b)

**Figure:**
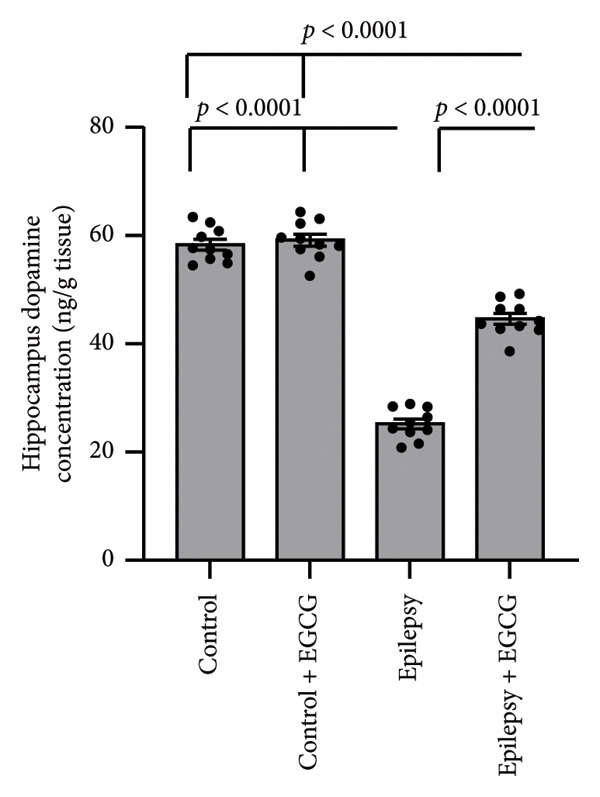
(c)

**Figure:**
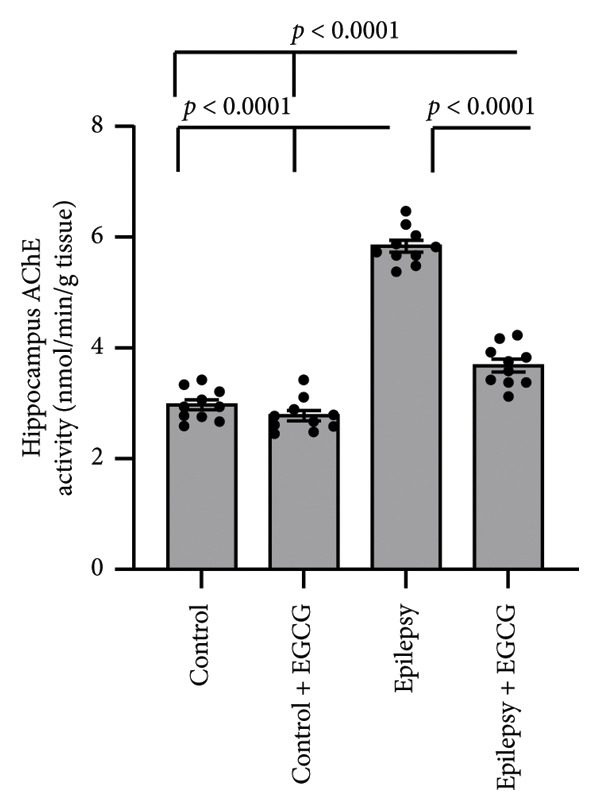
(d)

### 3.2. Effect of EGCG on Epilepsy‐Induced Alterations in Hippocampal Tissue Structure

**Figure 2:**
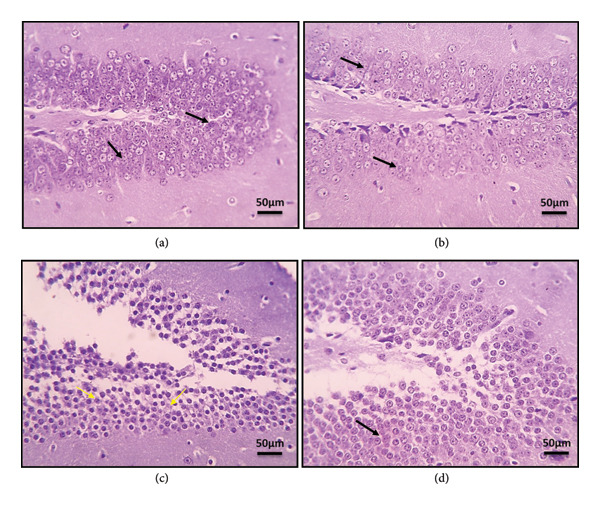
Microscopic pictures of the cresyl violet–stained sections from DG showing the average staining intensity of Nissl granules (black arrows) in the control group (a) and the control group treated with EGCG (b) sections from the DG of the epilepsy group (c) showed markedly decreased staining intensity of Nissl granules (yellow arrows). Sections from the DG of the epilepsy group treated with EGCG (d) show the slightly increased staining intensity of Nissl granules (black arrows). Scale bar 50 μm. DG, dentate gyrus; EGCG, Epigallocatechin‐3‐gallate.

In the present research, cresyl violet–stained microscopic images were used to examine the sections of the dentate gyrus. The analysis revealed that the control group exhibited an average staining intensity of Nissl granules. However, the sections from the dentate gyrus of the epilepsy group displayed a notable decrease in the staining intensity of Nissl granules, as indicated by a thin black arrow. Interestingly, the sections from the dentate gyrus of the epilepsy group treated with EGCG showed a slight increase in the Nissl granule staining intensity, as indicated by a thin black arrow (Figure 2).

### 3.3. Effect of EGCG on the Epilepsy‐Induced Downregulation of Nrf2

**Figure 3:**
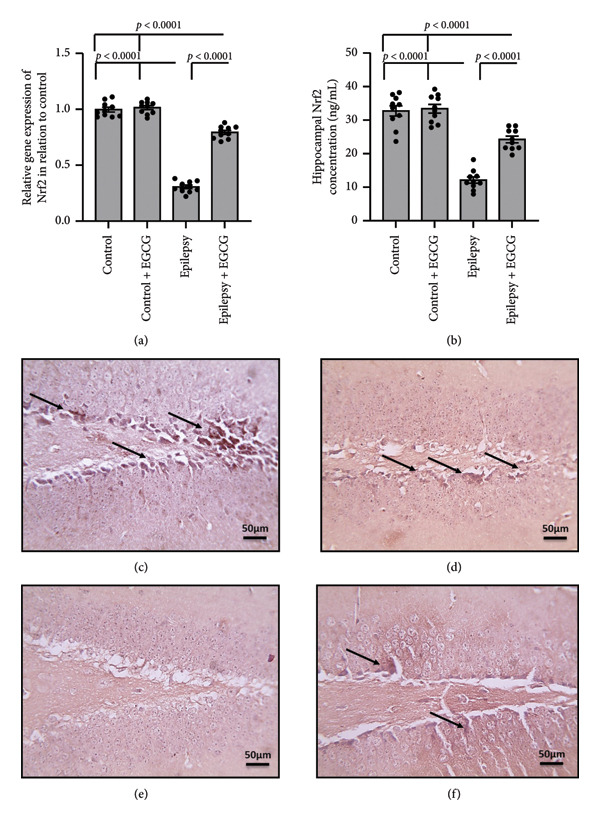
Effect of epilepsy and 20 mg/kg EGCG on Nrf2 gene expression (a) and protein level (b) brain cortex sections stained with anti‐Nrf2 antibodies in the control group (c), control group treated with EGCG (d), epilepsy group (e), and epilepsy treated with EGCG (f). EGCG, Epigallocatechin‐3‐gallate; Nrf2, Nuclear Factor Erythroid 2–related factor‐2.

In Figure 3, involving rats with epilepsy, researchers analyzed hippocampal tissue and observed a 69% decrease in Nrf2 gene expression, a crucial transcription factor involved in the body’s defense against oxidative stress. Furthermore, there was a 63% reduction in Nrf2 protein levels in the hippocampal tissues of epileptic rats compared with the control group. The researchers also examined micro‐images of hippocampal tissue stained with anti‐Nrf2 antibodies and observed a significant decrease in the stained area in epilepsy rats compared to the control group. However, administering EGCG, a potent antioxidant found in green tea, to epileptic rats significantly reversed these effects. This reversal was demonstrated in Figure 3 of the study, indicating a potential therapeutic role for EGCG in modulating Nrf2 expression and activity in the context of epilepsy.

### 3.4. Effect of EGCG on the Epilepsy‐Induced Downregulation of HO‐1

Based on the research findings, the expression of HO‐1 genes was significantly reduced by 56% in rats with epilepsy compared with the control group. Moreover, HO‐1 protein levels in the hippocampus were reduced by 62% in the epilepsy group compared with the control group. Nevertheless, administering EGCG to epilepsy‐afflicted rats increased HO‐1 gene expression and protein levels. Despite this increase, both gene expression and protein levels remained lower than those in the control group, as illustrated in Figure 4.

Figure 4Effect of epilepsy and 20 mg/kg EGCG on the gene expression of HO‐1 (a) and its protein level (b). EGCG, Epigallocatechin‐3‐gallate; HO‐1, Heme Oxygenase‐1.

**Figure:**
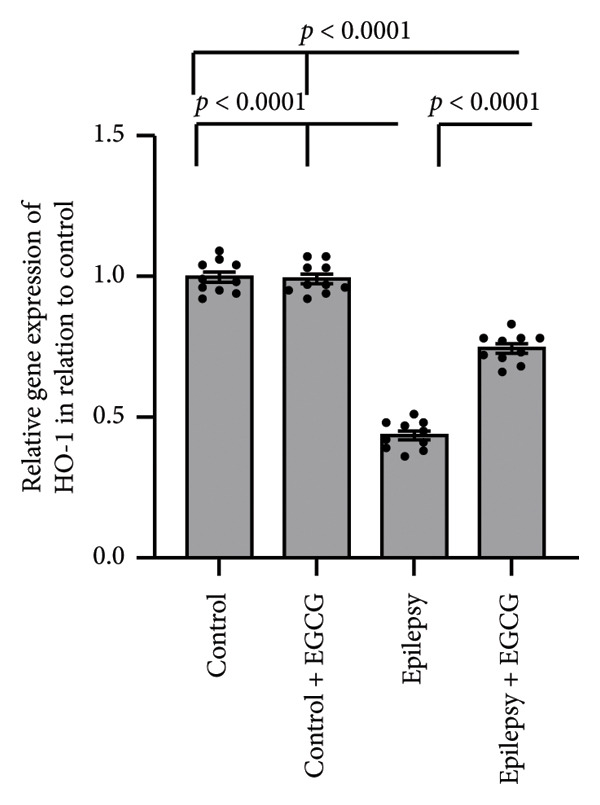
(a)

**Figure:**
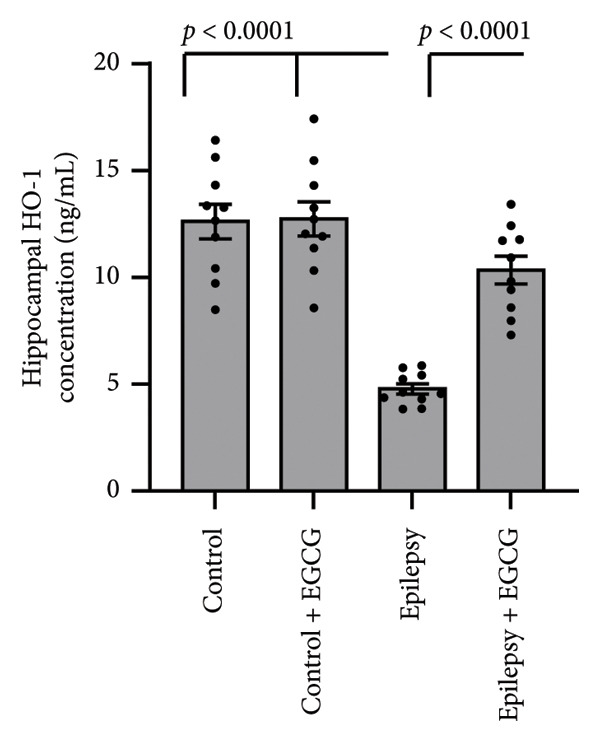
(b)

### 3.5. Effect of EGCG on Epilepsy‐Induced Elevation in NFκB

**Figure 5:**
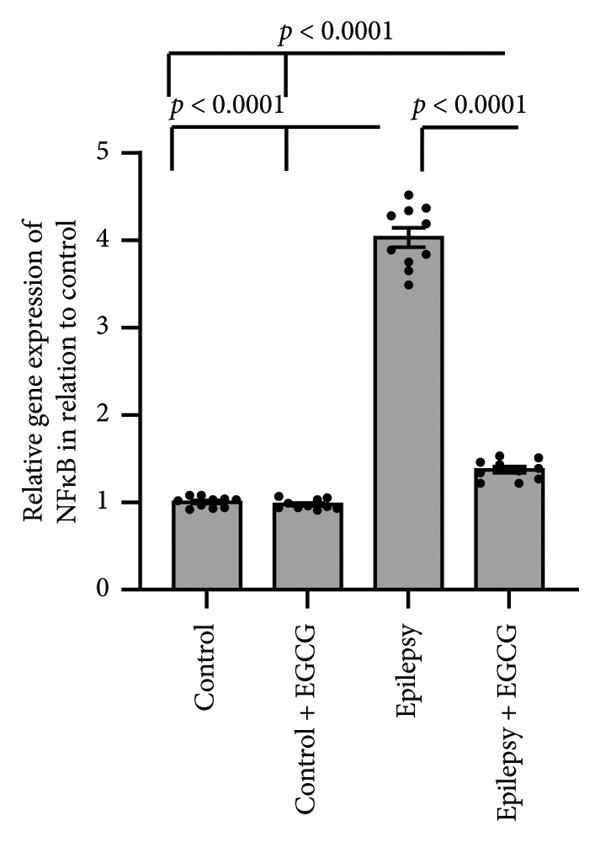
Effect of epilepsy and 20 mg/kg EGCG on the gene expression of NFκB. EGCG, Epigallocatechin‐3‐gallate; NFκB, nuclear factor κB.

The results of the study demonstrate that epilepsy leads to a substantial upregulation of NFκB gene expression, showing a 4.03‐fold increase compared to the control group in rats. Interestingly, treatment with EGCG reduced NFκB expression in the epilepsy group; however, it remained lower than in the control group, as illustrated in Figure 5.

### 3.6. Effect of EGCG on Epilepsy‐Induced Activation of the Inflammatory Pathway

In our research on epilepsy development, we concentrated on how EGCG influences the expression of inflammatory proteins TNF‐α and IL‐1β. Our findings indicated a marked increase in TNF‐α and IL‐1β gene expression by 3.17‐ and 3.26‐fold, respectively, in epileptic rats compared with the control group. Furthermore, we observed a 3.46‐ and 3.01‐fold increase in hippocampal TNF‐α and IL‐1β protein concentrations, respectively, in epileptic rats compared to control subjects. However, administering EGCG to epileptic rats resulted in a significant reversal of these changes (Figure 6).

Figure 6Effect of epilepsy and 20 mg/kg EGCG on the gene expression of TNF‐α (a) and IL‐1β (c), as well as protein levels of TNF‐α (b) and IL‐1β (d). EGCG, Epigallocatechin‐3‐gallate; IL‐1β, Interleukin‐1 β; TNF‐α, tumor necrosis factor‐α.

**Figure:**
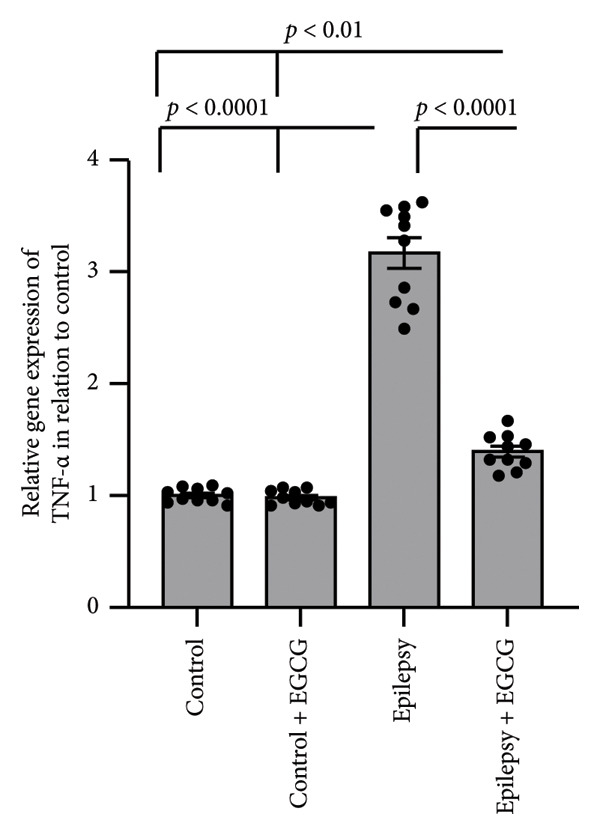
(a)

**Figure:**
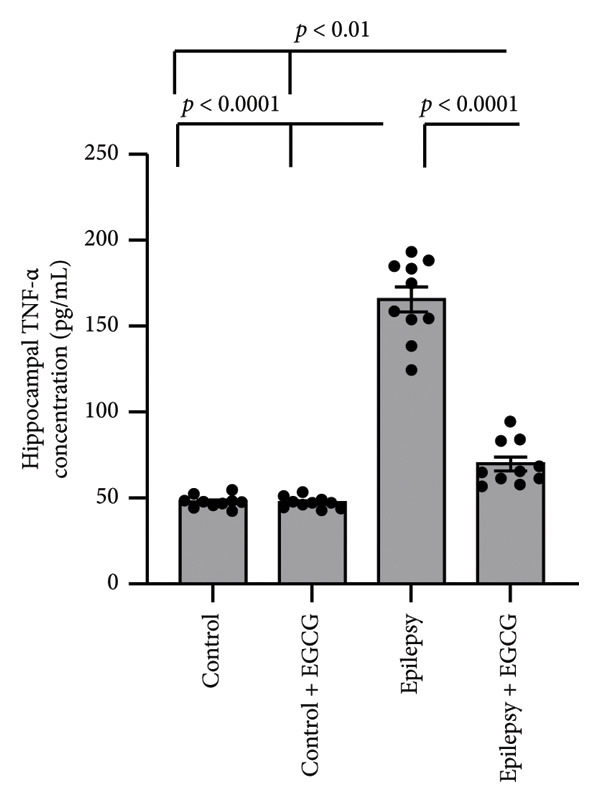
(b)

**Figure:**
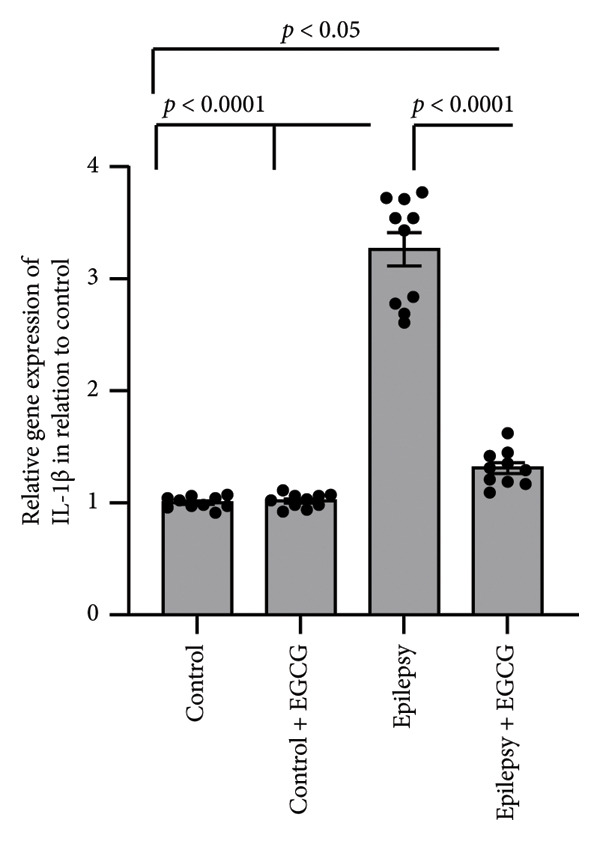
(c)

**Figure:**
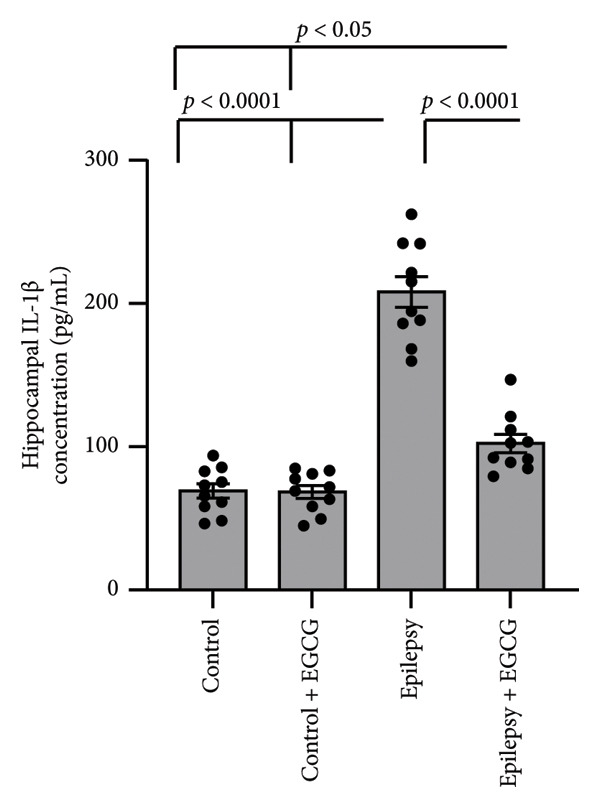
(d)

### 3.7. Effect of EGCG on Epilepsy‐Induced Activation of the Apoptotic Pathway

In the context of epilepsy progression, our research concentrated on how EGCG affects the balance between the anti‐apoptotic protein BCL2 and the pro‐apoptotic protein BAX. Our findings suggest that epilepsy results in a 66% decrease in BCL2 gene expression and a 3.34‐fold increase in BAX gene expression relative to the control group. Examination of protein levels in the hippocampal tissue of epileptic rats showed a 57% reduction in BCL2 and a 3.62‐fold increase in BAX when compared to the control group. Nevertheless, treatment with EGCG in epileptic rats successfully reversed these changes, as evidenced by the upregulation of BCL2 and the downregulation of BAX. As a result, EGCG demonstrated a protective role in hippocampal tissues by inhibiting the apoptotic pathway (Figure 7).

Figure 7Effect of epilepsy and 20 mg/kg EGCG on the gene expression of BCL2 (a) and BAX (c), as well as the protein levels of BCL2 (b) and BAX (d). BCL2, B‐Cell Lymphoma 2; BAX, BCL2‐associated X; EGCG, Epigallocatechin‐3‐gallate.

**Figure:**
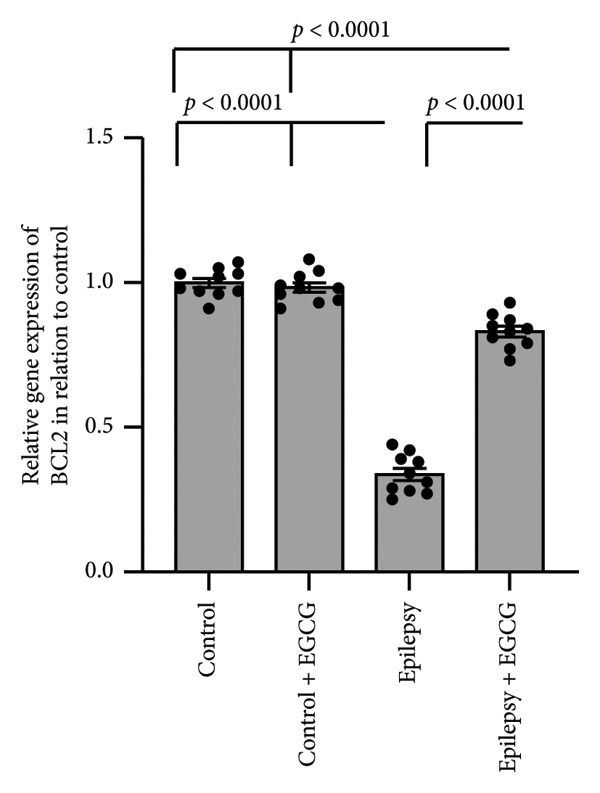
(a)

**Figure:**
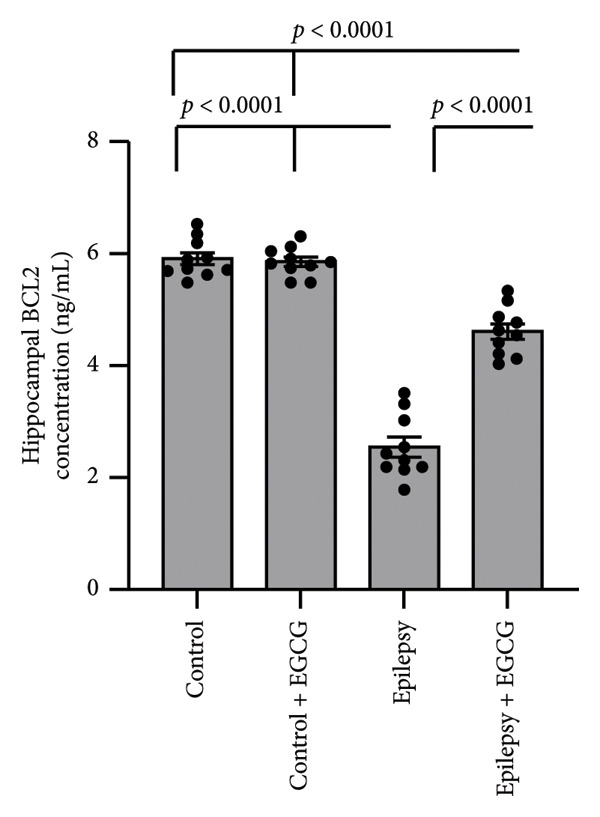
(b)

**Figure:**
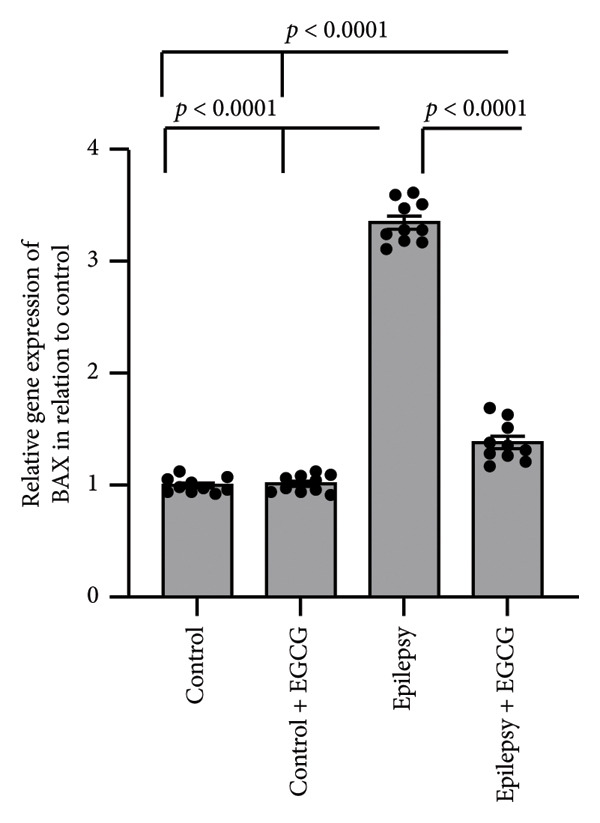
(c)

**Figure:**
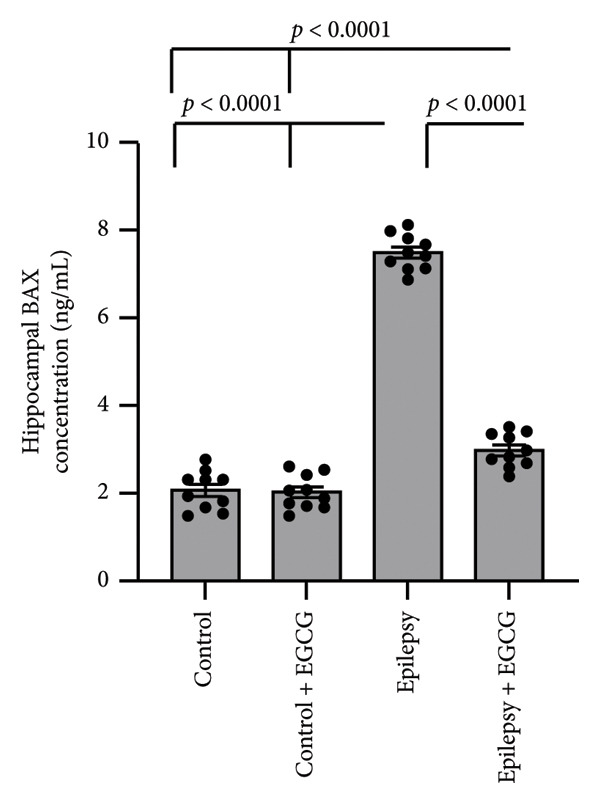
(d)

## 4. Discussion

Epilepsy is a severe neurological disorder characterized by unpredictable and recurrent seizures resulting from sudden, abnormal electrical discharges in the brain, which can interfere with normal brain functions. The symptoms can be profound, including the abrupt loss of consciousness, severe convulsions, involuntary movements, and unsettling sensory distortions or emotional fluctuations. This condition affects nearly 50 million individuals globally and poses an even greater challenge in low‐ and middle‐income countries, where its prevalence and risk of onset are significantly higher [18]. Our study employed the PTZ model in rats to investigate epilepsy. The administration of PTZ led to an increased frequency and intensity of seizures, correlated with a decrease in dopamine levels and an increase in AChE activity. Moreover, the examination of brain sections stained with cresyl violet revealed a significant reduction in the Nissl granule staining intensity, indicating neuronal alterations associated with the disorder. In parallel, EGCG is a potent polyphenol predominantly present in green tea, recognized for its antioxidant and anti‐inflammatory properties. In our study, we observed that the administration of EGCG to epileptic rats reduced both the frequency and intensity of seizures, alongside the restoration of normal dopamine and AChE concentrations. Furthermore, histological analysis of the dentate gyrus in the EGCG‐treated group showed a modest increase in the Nissl granule staining intensity. These findings suggest that EGCG may serve as a viable therapeutic option for managing epilepsy. The subsequent phase of our research will focus on elucidating the mechanism of action of EGCG in epilepsy‐induced rat models.

During epileptic seizures, a profound and pathological increase in neuronal excitability occurs, resulting in heightened and excessive neuronal firing. This surge in activity not only amplifies the frequency of action potentials but also leads to synchronized discharges across neural networks, contributing to the seizure phenomena. As a consequence of this hyperactivity, there is a significant escalation in cerebral oxygen consumption, which is essential for sustaining the intensive metabolic demands of the hyperactive neurons [19]. This augmented demand for oxygen disrupts the normal functioning of the mitochondrial respiratory chain, compromising its ability to efficiently produce ATP. The inefficiency of mitochondrial respiration leads to an alarming accumulation of ROS, including superoxide radicals and hydrogen peroxide. These ROS can cause oxidative stress, damaging cellular components, including lipids, proteins, and DNA, further exacerbating neuronal dysfunction and potentially contributing to the progression of epilepsy and related neurological disorders [20]. Nrf2 is a pivotal transcription factor that plays a significant role in cellular defense mechanisms by orchestrating the expression of a variety of antioxidant and detoxifying enzymes. Among these enzymes, HO‐1 is particularly notable due to its essential function in the catabolism of heme [21]. HO‐1 facilitates the degradation of heme into three primary products: biliverdin, which can be further converted to bilirubin, free iron, and carbon monoxide. This process is critical for maintaining cellular homeostasis and providing a defense against oxidative stress, which can lead to cellular injury if not properly regulated. The interaction between the Nrf2 and HO‐1 signaling pathways has garnered attention in the context of neurological disorders, particularly epilepsy. During epileptic seizures, there is an increase in oxidative stress and inflammation, both of which contribute significantly to neuronal damage and cell death. Nrf2 activation leads to the upregulation of HO‐1, which can help alleviate oxidative stress by enhancing heme degradation and promoting the production of antioxidant molecules [22]. Recent research indicates that the Nrf2‐HO‐1 axis plays a critical neuroprotective role in epilepsy. This pathway appears to be instrumental in counteracting the detrimental effects of oxidative stress and inflammation, both of which are known to exacerbate neuronal damage during seizures. By supporting the body’s natural defense mechanisms, activating this axis may significantly alleviate seizure‐induced neurotoxicity. By bolstering brain cells’ resilience against the biochemical damage that often accompanies seizures, the Nrf2/HO‐1 signaling pathway holds promise for protecting neuronal integrity and enhancing overall brain health and cognitive performance. This is particularly important as neurodegeneration can lead to long‐term cognitive impairments following seizure activity [23]. Consequently, targeting the Nrf2/HO‐1 signaling pathway is emerging as a compelling strategy for developing novel therapeutic interventions aimed at safeguarding the brain from the harmful consequences of epilepsy and related neurodegenerative disorders. Researchers are increasingly focusing on pharmacological agents and lifestyle modifications that may activate this pathway, opening the door to innovative treatments that could revolutionize care for individuals with these conditions.

Our research revealed a significant decrease in the expression levels of both Nrf2 and HO‐1 in rats subjected to epilepsy. Notably, this reduction in expression was effectively reversed by EGCG administration. In addition, prior research has shown that EGCG can potentially mitigate neuronal cell death. This is achieved by modulating several critical factors, including Glutathione Peroxidase 1 and the phosphorylation status of NFκB, as well as influencing mitochondrial dynamics. This specific effect of EGCG was observed in the hippocampal region of the rat brain following an induced state of status epilepticus, highlighting the therapeutic potential of EGCG in epilepsy management and its impact on neuroprotection [24].

ROS play a crucial role in the pathophysiology of neuroinflammation by promoting the release of pro‐inflammatory cytokines, including IL‐1β and TNF‐α. The release of these cytokines amplifies the inflammatory response, contributing to a cascade of neuroinflammatory processes that not only exacerbate mitochondrial dysfunction but also impair neuronal function [25]. This creates a detrimental feedback loop characterized by heightened oxidative stress, resulting in significant neuronal damage. The resultant neuronal impairment can trigger hyperexcitability, culminating in the manifestation of epileptic seizures. This self‐perpetuating cycle underscores the intricate interplay between oxidative stress, neuroinflammation, and seizure activity, highlighting potential therapeutic targets for intervention [26]. NFκB, TNF‐α, and IL‐1β are key mediators of inflammation and immune responses in the body. This line of research highlights the critical roles of these inflammatory mediators in the neuroinflammatory processes that accompany epileptic seizures. NFκB constitutes an essential transcription factor that orchestrates the expression of various pro‐inflammatory genes. Upon activation, particularly by cytokines, NF‐kB translocates to the cell’s nucleus, binds to specific DNA sequences, and facilitates the transcription of genes that promote inflammation. These cytokines are known to be pivotal in modulating neuronal excitability and synaptic plasticity, which can contribute to increased seizure susceptibility [27]. TNF‐α is produced primarily by activated macrophages and is vital in mediating systemic inflammation. It enhances the inflammatory response and affects various cellular processes, including survival, proliferation, and apoptosis, thereby influencing tissue health or disease [28]. Similarly, IL‐1β is a potent pro‐inflammatory cytokine secreted by various cells, including macrophages and dendritic cells. It contributes to the inflammatory process by inducing the expression of adhesion molecules that promote leukocyte recruitment to sites of inflammation [29]. IL‐1β, in particular, has been linked to glial cell activation and the subsequent release of additional inflammatory mediators, creating a feedback loop that can exacerbate neuronal hyperexcitability. Similarly, TNF‐α has been recognized for its dual role in promoting neuronal survival and influencing neuronal excitability, depending on its concentration and the context of its action [30]. In summary, the relationship between these inflammatory cytokines and epilepsy is multifaceted. Understanding the interplay between NFκB, IL‐1β, and TNF‐α not only sheds light on the mechanisms underlying epilepsy but also opens avenues for identifying potential therapeutic targets. By manipulating these pathways, it may be possible to develop novel interventions aimed at reducing seizure frequency and improving outcomes for individuals suffering from epilepsy, as evidenced by findings derived from rat models.

In our study, we observed a marked increase in the expression levels of key inflammatory markers, specifically NFκB, IL‐1β, and TNF, in rats diagnosed with epilepsy. This elevation suggests a potential link between these markers and the pathophysiology of epilepsy. However, after administering EGCG, we observed a significant reversal of NFκB, IL‐1β, and TNF overexpression. This finding is particularly noteworthy as it adds new insights into the therapeutic potential of EGCG for managing inflammation associated with epilepsy. Notably, there is a lack of prior research specifically examining the effectiveness of EGCG in mitigating elevated expression of these inflammatory markers in animal models of epilepsy.

In addition to their regulatory functions, cytokines can exhibit cytotoxic and pro‐apoptotic effects, contributing to the processes of cell survival and death within the central nervous system. One of the remarkable features of cytokines is their ability to cross the BBB, enabling bidirectional communication between the peripheral immune system and the CNS. Monocytes and macrophages are stimulated by cytokines to release further pro‐inflammatory mediators, thereby amplifying the neuroinflammatory response, leading to increased apoptosis and neuronal loss [31]. BCL2 and BAX are pivotal proteins in the BCL2 family, a diverse group of regulators that play essential roles in cell death, particularly through apoptosis. BCL2 serves a protective role in cellular functioning by inhibiting apoptosis, thereby ensuring cell survival under various conditions. On the other hand, BAX is recognized as an apoptotic activator that promotes the onset of apoptosis in response to cellular stressors or damage. Both proteins are integral to maintaining the delicate balance of cell life and death, which is critical for overall tissue homeostasis [32]. In the context of epilepsy, patients often endure poorly controlled seizures that can lead to extended seizure episodes. This chronic condition can lead to cumulative cellular damage and, over time, the loss of neurons. However, recent research has shed light on the multifaceted roles of BCL2 family proteins beyond their traditional functions in apoptosis. It has been revealed that these proteins also possess neuroprotective properties, which can attenuate the symptoms of epilepsy. They achieve this by modulating neuronal excitability, which is a key factor in the manifestation and propagation of seizures [33]. Specifically, BCL2 family proteins regulate the activity of various ion channels and transporters at the neuronal membrane. These ion channels play a critical role in regulating the flow of ions—such as sodium, potassium, calcium, and chloride—in and out of neurons, thereby impacting their excitability. For instance, alterations in ion flow can lead to hyperexcitability, which is a hallmark of seizure activity. By fine‐tuning these ionic currents, BCL2 family proteins can stabilize neuronal firing rates and reduce the likelihood of seizures [34]. Furthermore, BCL2 family proteins are implicated in the regulation of neurotransmitter release, which is essential for neuronal communication. Neurotransmitters are chemical messengers that transmit signals across synapses, and their precise release hinges on the coordinated activity of various cellular mechanisms. By modulating these processes, BCL2 proteins can affect not only synaptic function but also overall brain circuitry, further influencing neuronal excitability and seizure thresholds [35]. Therefore, a deeper understanding of the multiple roles of BCL2 family proteins in epilepsy holds significant promise for the development of innovative therapeutic strategies. Targeting these proteins could pave the way for novel interventions that not only mitigate seizure activity but also promote neuronal health and resilience [36].

In our study, we examined the intricate relationship between epilepsy and the expression of key apoptosis‐related proteins. Our findings revealed a significant reduction in BCL2 gene expression and protein levels, and a notable increase in BAX levels, indicating an imbalance that likely contributed to the enhanced neuronal cell death associated with this neurological disorder. Furthermore, we investigated the potential therapeutic effects of EGCG. Remarkably, our results demonstrated that treatment with EGCG led to a substantial increase in BCL2 expression, effectively restoring its levels to those comparable to those in nonepileptic conditions. At the same time, EGCG treatment decreased BAX levels, helping rebalance the pro‐apoptotic and anti‐apoptotic signals within cells. Notably, our study is the first to demonstrate EGCG’s ability to restore the normal balance between BCL2 and BAX in the context of epilepsy, paving the way for further research into its potential as a treatment option for managing this challenging condition.

Although EGCG has shown potential therapeutic advantages for epilepsy treatment, it is vital to consider the considerable limitations associated with the research methods employed, including the lack of data from human subjects, the short duration of the studies, and the failure to conduct dose–response analyses. Another crucial limitation is reliance on rat models, as rats have distinct metabolic processes and produce different drug metabolites that do not correspond to those made in humans. Consequently, caution is warranted when evaluating the study’s findings for potential human applications.

Despite these limitations, our research provides valuable insights into the potential therapeutic application of EGCG for epilepsy, laying a foundation for future clinical trials involving human subjects. First, EGCG exhibits significant antioxidant, anti‐inflammatory, and anti‐apoptotic properties. Secondly, it reduced the severity of epilepsy by targeting crucial mechanisms involved in the disease. Third, EGCG is a safe natural compound with low toxicity levels, as it was found to elicit toxicity in the range from 500 to 1500 mg/kg in rats. When translated into human equivalents, this corresponds to a range of approximately 30 to 90 mg/kg, based on the average daily caloric requirements of mice (12 kcal) compared to those of humans (2000 kcal). While current research shows promise, human clinical trials are necessary to confirm the effectiveness and safety of this approach for patients with sciatic nerve injuries. Currently, there are no results from clinical trials specifically focused on using EGCG to treat epilepsy in the available search results. Until these clinical trials are completed, individuals can still benefit from the health benefits of EGCG by consuming foods rich in it. To put this into perspective regarding green tea consumption, the toxic dosage corresponds to an estimated intake of about 10.5 to 32 cups of green tea per day, assuming that each cup is brewed using 2.5 g of green tea leaves steeped in 250 mL of water [37]. By incorporating herbal products rich in EGCG into their daily diets, individuals can achieve meaningful doses. Consequently, the dosages used in our study could realistically be approximated by consuming approximately one cup of green tea per day.

## 5. Conclusion

EGCG has shown significant neuroprotective effects in the context of epilepsy. These protective mechanisms manifest not only in behavioral improvements but also in the restoration of normal neuronal structures. The neuroprotective properties of EGCG are primarily linked to its ability to enhance the body’s antioxidant defenses. This is achieved by upregulating critical signaling proteins, such as Nrf2 and HO‐1. Moreover, EGCG has been shown to possess anti‐inflammatory properties, largely by downregulating pro‐inflammatory pathways. This includes the reduced expression of NFκB, TNF‐α, and IL‐1β. Additionally, EGCG contributes to cell survival by influencing apoptosis. It promotes the downregulation of BAX, a pro‐apoptotic protein, while simultaneously upregulating BCL2, an anti‐apoptotic protein. This shift in the balance between pro‐ and anti‐apoptotic signals helps reduce apoptosis, thereby preserving neuronal health and functionality.

## Conflicts of Interest

The authors declare no conflicts of interest.

## Funding

The current research did not receive any type of funding.

## Data Availability

Data are available from the corresponding author upon suitable request.
